# Thinking Beyond Bacterial Infection: A Case of Cutaneous Blastomycosis

**DOI:** 10.7759/cureus.21634

**Published:** 2022-01-26

**Authors:** Mehakmeet Bhatia, Vivek Kak, Parth Patel, Alexander Slota

**Affiliations:** 1 Internal Medicine, Henry Ford Health System, Detroit, USA; 2 Infectious Disease, Henry Ford Allegiance Health, Jackson, USA; 3 Hematology/Oncology, Henry Ford Health System, Detroit, USA

**Keywords:** itraconazole, wisconsin, pulmonary blastomycosis, biopsy, cutaneous blastomycosis, disseminated blastomycosis

## Abstract

Blastomycosis is caused by *Blastomyces dermatitidis*, a dimorphic fungus that primarily causes pulmonary disease. Cutaneous blastomycosis is infrequent and tends to be misdiagnosed given its similar presentation to other cutaneous fungal infections and malignancies. A 51-year-old woman presented with a two-month history of disfiguring nasal lesions. The patient had a past medical history of cervical cancer which was currently in remission. Social history was significant for frequent travel throughout the United States as a truck driver, including the Midwest. The patient had a non-purulent verrucous plaque on her right nare, which was painless and mildly pruritic. Superficial cultures grew *Enterococcus faecalis*, prompting treatment with oral cephalexin and topical mupirocin. Given no relief, the patient was started on clindamycin followed by Augmentin. Both treatments were unsuccessful.

The lesion was then biopsied and fungal cultures were sent. The biopsy showed broad-based budding yeast surrounded by pseudoepitheliomatous hyperplasia, and cultures grew *Blastomyces dermatitidis*. The patient was initiated on 200 mg itraconazole thrice daily for the first three days, followed by 200 mg itraconazole twice daily for the next 12 months. She showed notable improvement within a month.

This patient was initially misdiagnosed with bacterial infection due to superficial cultures, which were likely a contaminant. It was only after a biopsy that the patient was accurately diagnosed. Besides bacterial infection, cutaneous blastomycosis is often confused with coccidioidomycosis, mycobacterial infection, or squamous cell carcinoma. In patients such as ours who are presenting with persistent facial lesions in the setting of frequent travel history, fungal etiologies should be high on the differential. A biopsy and fungal cultures should be sent at the outset for accurate diagnosis and treatment.

## Introduction

Blastomycosis is caused by a dimorphic fungus, *Blastomyces dermatitidis*, that mainly causes pulmonary disease [1,2]. Cutaneous blastomycosis is infrequent. The diagnosis is often delayed because of low clinical suspicion and similar presentation to bacterial or other fungal infections, such as coccidioidomycosis or squamous cell carcinoma [1-5].

## Case presentation

**Figure 1:**
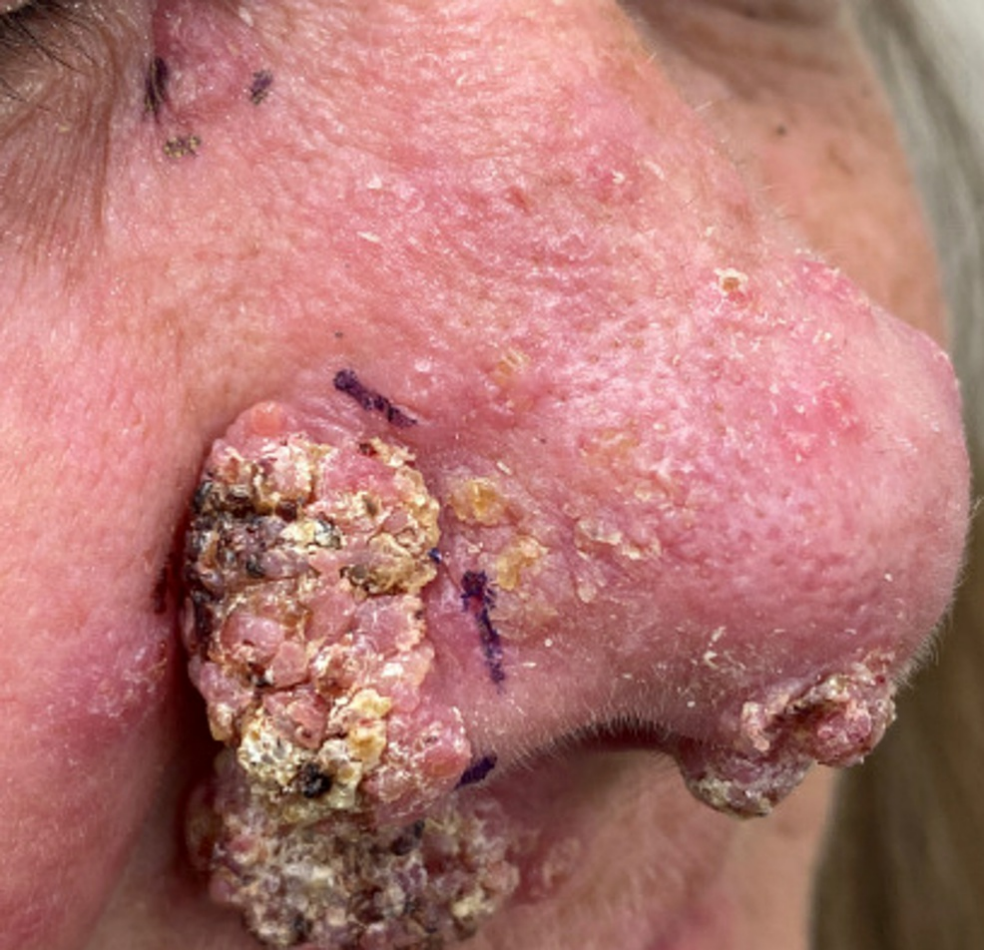
Verrucous plaque localized to right nare. The lesion was primarily exophytic, non-purulent, and painless.

A 51-year-old woman presented with a two-month history of disfiguring nasal lesions (Figure 1). The patient had a past medical history of cervical cancer. She had undergone chemotherapy several years ago but was currently in remission, and therefore was no longer immunocompromised. Social history was significant for frequent travel throughout the United States as a truck driver, including the Midwest. The patient had a non-purulent verrucous plaque on her right nare, which was painless and mildly pruritic. Superficial cultures grew *Enterococcus faecalis*, prompting treatment with oral cephalexin and topical mupirocin. Given no relief, the patient was started on clindamycin followed by Augmentin. Both treatments were unsuccessful. 

The lesion was then biopsied and fungal cultures were sent. Pathologic examination revealed broad-based budding yeast surrounded by pseudoepitheliomatous hyperplasia on periodic acid Schiff (PAS) stain, and cultures grew *Blastomyces dermatitidis*. The patient underwent CT chest without contrast to evaluate for a pulmonary source of blastomycosis, which was subsequently negative for any infiltrates. Upon further chart review and history obtained from the patient, it was determined that the patient had not been hospitalized for a pneumonia. The patient denied any laboratory or autopsy exposure to infected needles. The patient did have a pet dog, however, denied any animal bites and admitted to her dog being healthy. However, as part of her job as a truck driver, the patient was exposed to dust and soil particles. At this point, the patient’s history was suggestive of either primary inoculation through soil or secondary disease resulting from a pulmonary source where the patient was asymptomatic. The patient also underwent ophthalmologic examination which was negative for any ocular invasion.

**Figure 2:**
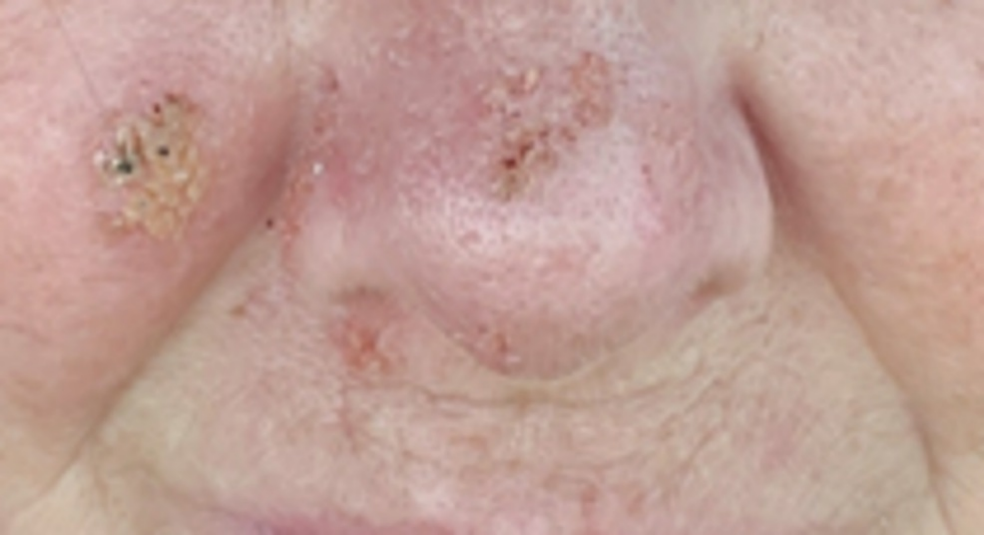
Significant improvement noted after one month of treatment with oral itraconazole.

The patient was initiated on 200 mg itraconazole thrice daily for the first three days, followed by 200 mg itraconazole twice daily for the next 12 months. For the first month after treatment initiation, the patient followed weekly to evaluate liver function tests, and biweekly for serum itraconazole levels. The patient was 100% compliant, had normal liver function tests and therapeutic serum itraconazole levels throughout her treatment. The patient alternated between telephone and in-person visits with infectious disease department at Henry Ford Allegiance Health. She showed notable improvement within a month (Figure 2). The patient will be close to the end of her treatment after 12 months at the end of January 2022. 

## Discussion

Within the United States, blastomycosis is primarily found in the Midwest, south-central, and south-eastern regions, including the Ohio-Mississippi and Saint Lawrence River valleys and the Great Lakes region [1,2]. The distribution overlaps with histoplasmosis. However, histoplasmosis tends to localize in caves rather than in the decaying vegetation and acidic soils where blastomycosis is found [2]. The risk of contracting blastomycosis is more significant with activities such as canoeing and fishing that disrupt the soil. Blastomycosis also affects immunocompetent hosts more frequently than other fungal infections, including coccidioidomycosis and histoplasmosis [2,6].

Blastomycosis most commonly presents with pulmonary symptoms after inhalation of blastomycosis spores or conidia. However, in up to 50% of cases, the patients are asymptomatic or develop subclinical symptoms that resolve spontaneously. Therefore, the disease often tends to be underdiagnosed [1]. Around 25-40% of the patients develop disseminated disease, most commonly manifesting as cutaneous findings [1,2,6]. The mode of dissemination is most often hematogenous or through lymph nodes from a pulmonary source [7]. However, cutaneous disease can present itself in the absence of overt pulmonary disease as shown in published case reports [6,7]. Cutaneous blastomycosis through primary inoculation is rare. The most common sources of primary inoculation are traumatic exposure through soil disrupting activities, animal bites (for instance dogs and cats), and laboratory of autopsy exposure to infected specimens [6,7]. Our patient did not report any specific trauma or pulmonary symptoms in the past, however, was exposed to soil and dust frequently as part of her job as a truck driver. This was suggestive of either a primary pulmonary source where she was asymptomatic, or primary exposure through soil as part of her job.

Cutaneous blastomycosis, like pulmonary disease, has a broad spectrum of findings. It can present as ulcerations with heaped borders or as verrucous nodules, large verrucae, plaques, or keloids. The lesions are often painless and are primarily found in exposed areas, including head, neck, and extremities [1,2,6-8].

**Figure 3:**
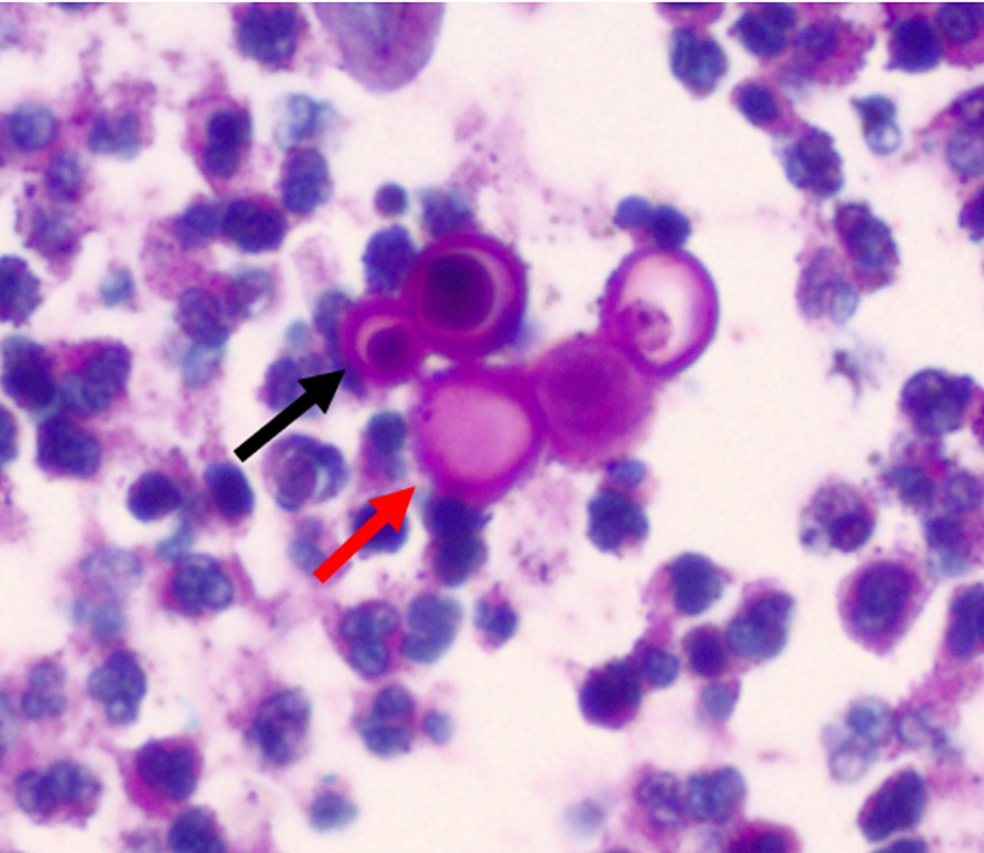
Round yeast cells (red arrow) with thick cell walls and broad-base buds (black arrow) as visualized on a PAS stain. PAS: periodic acid Schiff

**Figure 4:**
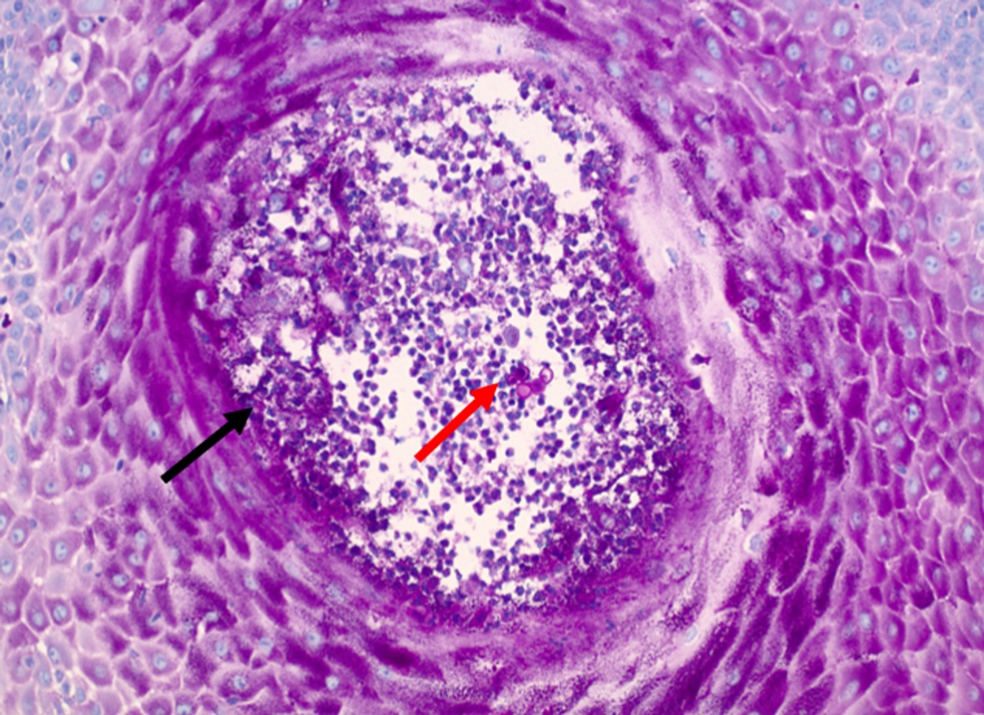
Pseudoepitheliomatous hyperplasia (black arrow). Round yeast cells with thick cell walls and broad-base buds (red arrow) as visualized on a PAS stain. PAS: periodic acid Schiff

Extrapulmonary disease is usually diagnosed with histopathologic examination of specimens with periodic acid Schiff (PAS) or methenamine silver stains [1,2,6-8]. For cutaneous lesions, it is recommended to obtain a deep tissue biopsy, including the entire thickness of the lesion. *Blastomyces dermatitidis* is characterized by round to oval, multinucleated yeast cells, with a broad-based bud and thick refractile cell walls (Figure 3) [1,2,6,8]. Pseudoepitheliomatous hyperplasia is also seen; however, it is not specific to blastomycosis and can resemble the pathological findings in squamous cell carcinoma (Figure 4) [2,9].

**Figure 5:**
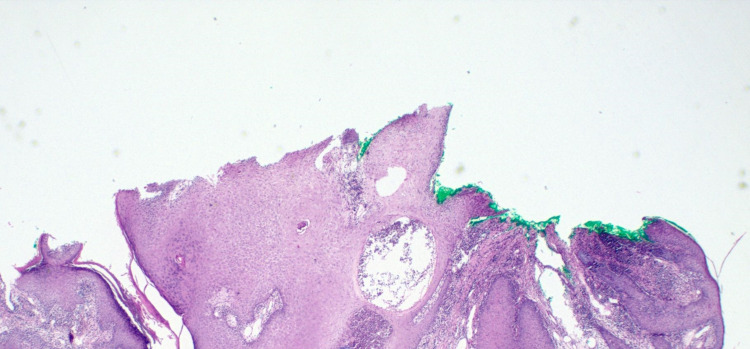
Hematoxylin-eosin stained section of verrucous lesion on right nare showing prominent inflammatory infiltrate in the dermis and pseudoepitheliomatous hyperplasia.

**Figure 6:**
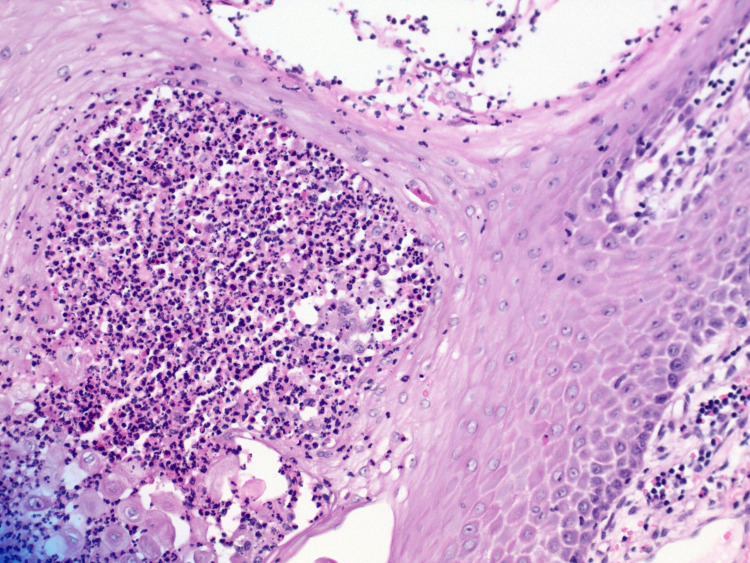
Hematoxylin-eosin stained section of verrucous lesion on right nare. A magnified version of slide in Figure 5 is showing a prominent inflammatory infiltrate in the dermis.

Hematoxylin-eosin stain can also be used to show dermal infiltrate and pseudoepitheliomatous hyperplasia (Figures 5, 6). A fungal culture is commonly used and takes up to five to 14 days to yield fungal growth. Notably, histopathology is less sensitive than culture; therefore, it is used in conjunction with culture. The urine antigen assay has cross-reactivity with *Histoplasma capsulatum*; likewise, serology is neither sensitive nor specific for blastomycosis [1,2,8].

Blastomycosis is often misdiagnosed as bacterial or fungal infection or even as malignancy such as squamous cell carcinoma [1-5]. The differential diagnoses also include leishmaniasis, lobomycosis, and even lupus vulgaris to name a few examples. Demographics and histology are key to an accurate diagnosis. Leishmaniasis is found primarily in Asia, Africa, America, and the Mediterranean region. Hematoxylin-eosin stain is used to identify early stages showing Leishman bodies which consists of parasites in cytoplasmic vacuoles in macrophages. While in later stages, granulomas are more common [10,11]. Lobomycosis is caused by the Lacazia loboi fungus [12]. This illness is seen primarily in America, with the highest prevalence in the Amazon basin. The illness mainly presents with keloid lesions however ulcerated lesions can be seen. Hematoxylin-eosin or silver stains can be used to visualize oval-shaped linked yeast structures forming a Rosario beads distribution [10]. Finally, lupus vulgaris is the most common manifestation of cutaneous tuberculosis. The illness is found most commonly in India [13,14]. Cutaneous form is primarily a result of reactivated tuberculosis infection caused by *Mycobacterium tuberculosis*. The illness presents as plaques or ulcerating lesions among other types. Staining with hematoxylin-eosin demonstrates granulomatous inflammation along with Langhans giant cells in dermis [13,14]. Cutaneous blastomycosis while presenting similarly, is markedly different in terms of histopathology as well as geographic distribution. 

Treatment depends on clinical judgment of disease severity. For mild to moderate disease, not involving the central nervous system, oral itraconazole is recommended, 200 mg thrice daily for the first three days, followed by the same dose once or twice daily for six to 12 months [9,13]. Serum itraconazole levels must be evaluated after at least two weeks of treatment. Itraconazole, like all azoles, can inhibit the P-450 system, leading to slowed metabolism and potential toxicity of other medications. Liver function tests should therefore be obtained periodically [9,13]. Itraconazole is also known to cause hypertension, edema, and hypokalemia and should be used cautiously in patients with congestive heart failure [1,2,8].

## Conclusions

Failure to respond to appropriate antibiotics should prompt a broader differential diagnosis beyond bacterial infection in cases of persistent dermatologic lesions. Cutaneous blastomycosis specifically should be suspected in patients with frequent travel in the Midwestern United States, despite the absence of recent respiratory infections. Primary inoculation, although rare, should be considered in patients with significant history of outdoor activities. In our patient, the initial diagnosis was hinged on a bacterial infection with *Enterococcus faecalis*. The patient’s travel history could have prompted an earlier investigation into fungal etiologies. However, it was not until the patient failed to respond to antibiotics that a biopsy and fungal cultures were sent, resulting in significant delay in treatment despite extensive spread of lesions. In order to prevent misdiagnosis and delays in treatment, fungal etiologies should be high on the differential; histopathologic specimen and fungal cultures should be sent at the outset for accurate diagnosis and treatment. In cases of fungal infection particularly blastomycosis, histology is key as it helps differentiate blastomycosis from various other infections and malignancies that have a similar presentation. Likewise, thorough exposure history and knowledge of other mimickers is just as important in prompt diagnosis and treatment of blastomycosis.
